# Black Phosphorus for Photonic Integrated Circuits

**DOI:** 10.34133/research.0206

**Published:** 2023-08-16

**Authors:** Mingxin Zhang, Lele Yang, Xiaoxuan Wu, Junjia Wang

**Affiliations:** National Research Center for Optical Sensors/communications Integrated Networks, School of Electronic Science and Engineering, Southeast University, 2 Sipailou, Nanjing 210096, China.

## Abstract

Black phosphorus gives several advantages and complementarities over other two-dimensional materials. It has drawn extensive interest owing to its relatively high carrier mobility, wide tunable bandgap, and in-plane anisotropy in recent years. This manuscript briefly reviews the structure and physical properties of black phosphorus and targets on black phosphorus for photonic integrated circuits. Some of the applications are discussed including photodetection, optical modulation, light emission, and polarization conversion. Corresponding recent progresses, associated challenges, and future potentials are covered.

## Introduction

In the past 30 years, information capacity has grown exponentially, and the large-scale deployment of high-speed communication networks has become an inevitable trend of the globalization process. These put forward higher demands for the technology that meets requirements in terms of capacity and consumption. Photonic integrated circuits are considered to be a promising candidate for next-generation communication systems owing to provide effective solutions with their advantages in transmission speed and energy efficiency. With its integrability with complementary metal oxide semiconductor fabrication, photonic integrated circuits play a crucial role in communication technology [1,2]. Despite substantial advances in high-efficiency and low-cost integrated photonic devices, such as photodetectors [3], sensors [4,5], modulators [6], and light emitters [7], large lattice mismatch and manufacturing complexity are still limiting factors for all integrated solutions.

**Fig. 1.**
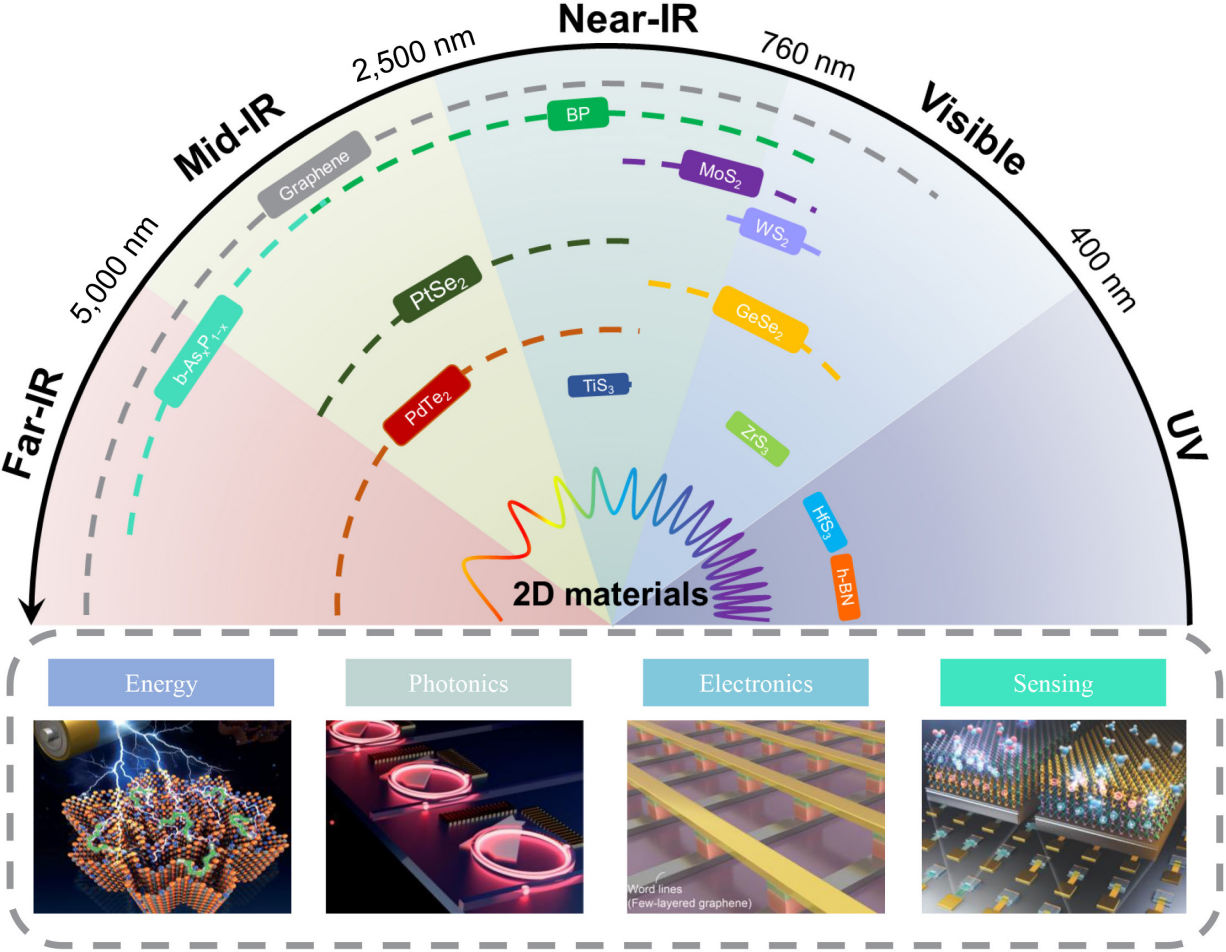
Comparison of the response range for various 2D materials and their potential applications. All images reproduced with permission: “Energy” from [129]. Copyright 2018 Wiley-VCH. “Photonics” from [130]. Copyright 2022 American Chemical Society. “Electronics” from [131]. Copyright 2023 Wiley-VCH. “Sensing” from [132]. Copyright 2020 Wiley-VCH. UV, ultraviolet.

Two-dimensional (2D) materials have drawn intense attention following the graphene was obtained by mechanical exfoliation of graphite in 2004 [8]. Since then, amazing progress has been made in this field, and they are widely applied in electronics, sensing, energy storage, and photonics (see Fig. 1). Numerous multifunctions and high-performance 2D materials integrated photonic components have been demonstrated by utilizing their advantages over optical and electronic properties, including high specific surface area, broadband absorption, strong light–matter interaction, and easy integration with silicon photonics [9–12]. Graphene, as a widely researched 2D material in recent years, has been demonstrated to be featured with outstanding mechanical strength, high light transmittance, superior carrier mobility, and high thermal conductivity [13–17]. However, optoelectronic applications of graphene are constrained by its zero bandgap, which leads to large dark current. Transition-metal dichalcogenides also present attractive properties, but their relatively wide bandgaps lead to very limited bandwidths and restricting their optoelectronic applications. In addition, the insertion loss is extremely high when the device is operating near the resonance wavelengths [18].

Black phosphorus (BP) has attracted considerable interest in catalytic systems, biomedicine, energy storage systems, catalytic systems, flexible sensors, and photonic integrated circuits over the past 10 years [19–26]. Although the first BP synthesis by Bridgman at 1.2 GPa and 200 °C can be traced back to the mid-1910 [27], there was not much interest in the material itself at that time. At the beginning of 2014, BP was restudied from the perspective of 2D materials [28] due to its exceptional physical characteristics including direct and tunable bandgap, relative high carrier mobility, and anisotropic properties [29–34].

Originally, BP was mostly synthesized by the high-pressure method [35]. Later, the synthesis processes of BP can be divided into bottom-up and top-down approaches. Bottom-up approaches like pulsed laser deposition and chemical vapor transport methods are commonly used for large-scale production [36–38]. However, the complex processes and high costs limit its feasibility to prepare the monolayer BP with high quality. Recently, Chen et al. [32] utilized controllable sustained vapor growth method to prepare large-scale BP thin film with high crystallinity and purity. The widely used top-down approaches for preparing few-layer BP with large lateral size are liquid exfoliation and mechanical exfoliation [39]. In order to improve the production rate, Zhang and co-workers [40] reported an intermediate-assisted grinding exfoliation to prepare various 2D layer materials, like monolayer or few-layer BP.

The BP bandgap is reduced from ∼2 eV for a monolayer to ∼0.3 eV for bulk materials because of the vertical quantum confinement effect [41,42]. As can be seen in Fig. 1, such a layer-tunable bandgap covers a wide electromagnetic spectral range from visible to mid-infrared (mid-IR) and bridges the gap between graphene and other 2D materials. Furthermore, BP bandgap is sensitive to a vertical electric field and can be efficiently tuned by electrical gating. Another important property is the saturable absorption, which may realize high-performance all-optical modulation. BP also preserves remarkable electrical performance, such as high carrier mobility up to 5,200 cm^2^ V^−1^ s^−1^, high on-off ratio exceeding 10^5^, and high saturation current in field-effect transistors [43–45]. The puckered arrangement of P atoms results in asymmetric crystal structure, which gives rise to its unique in-plane anisotropic electrical, thermal, and optical properties [46–48]. These extraordinary features make BP a very promising 2D material for diverse photonic applications, including wavelength converters [49], photodetectors [50], switches [51], modulators [52], and lasers [53]. BP is sensitive to some environmental factors such as oxygen and water [54,55]. Encapsulation [56], ligand surface coordination [57], doping [58], synchronous fluorination [59], covalent functionalization [60], and other strategies in the process of the fabrication have been presented to avoid degradation of BP and achieve excellent long-term stability. For example, Liu et al. [61] proposed a powerful short-distance transport growth method to enhance ambient stability by doping of various elements. Up to now, the most promising approach in practical application is to encapsulate with suitable materials such as aluminum oxide and packaging in inert atmosphere.

In this review, we briefly introduce the research advances of BP for photonic integrated circuits in recent years. First, the lattice structure and physical properties of BP are described. Subsequently, we focus on the applications of BP and their proceedings in photodetection, electro-optic (EO) modulation, all-optic modulation, light emission, and polarization conversion. Finally, based on BP current progress, current challenges and future prospects are highlighted.

## BP Structure and Properties

**Fig. 2.**
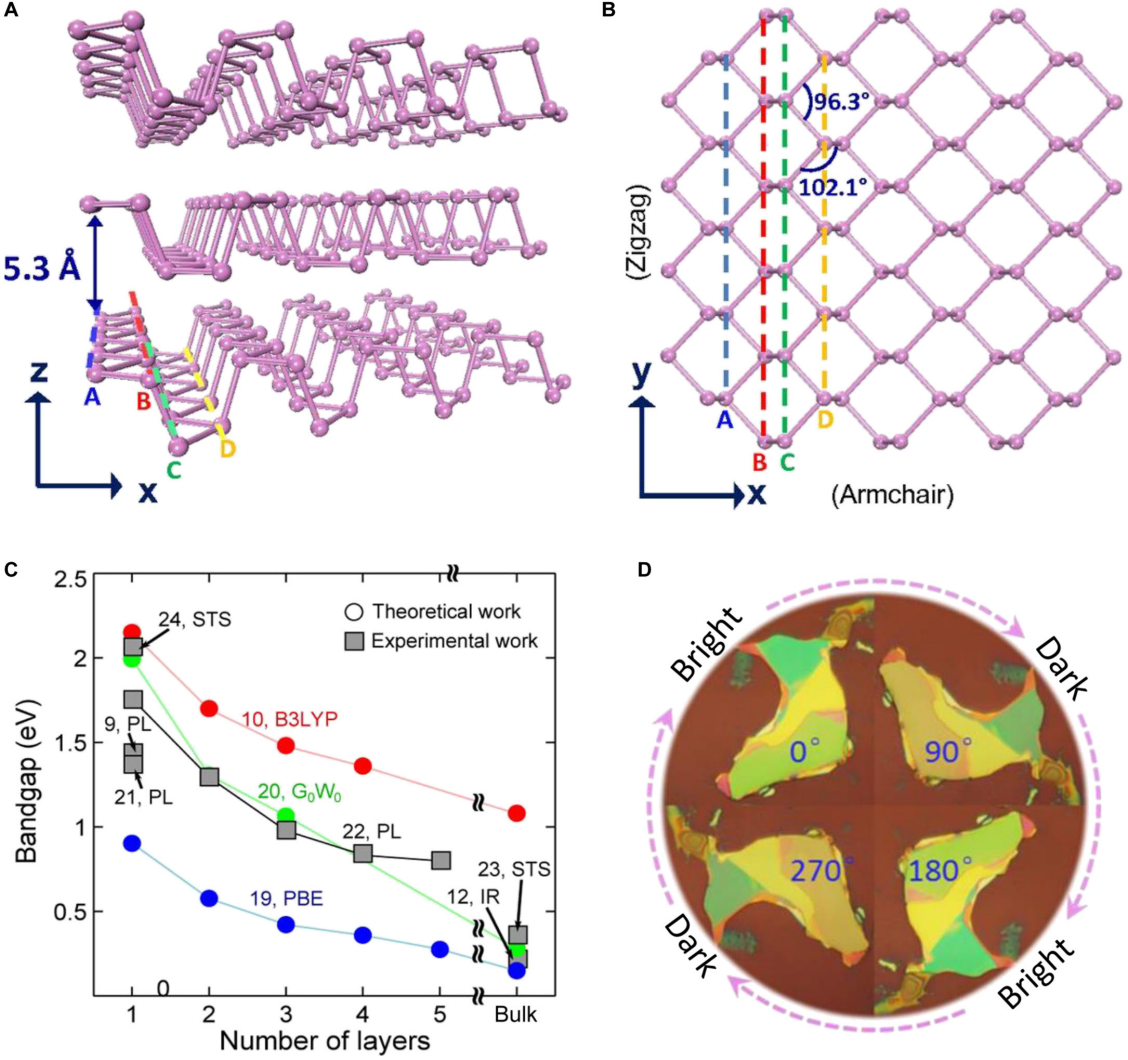
(A) Side view and (B) top view of the BP crystal structure. Reproduced with permission from [63]. Copyright 2015 National Academy of Sciences. (C) The BP bandgap as a function of layer numbers calculated by different methods. B3LYP, Becke, 3-parameter, Lee–Yang–Parr; G_0_W_0_, Green's function and a screened Coulomb interaction; PBE, Perdew–Burke–Ernzerho; STS, scanning tunneling spectroscopy. Reproduced with permission from [64]. Copyright 2015 American Chemical Society. (D) Optical images of few-layer BP with different rotation angles. Reproduced with permission from [79]. Copyright 2017 American Chemical Society.

Single-layer BP consists of a corner-sharing puckered honeycomb crystal structure where each phosphorus (P) atom is surrounded by three adjacent P atoms. Different from graphene, five outer shell electrons from a P atom are bonded to three outer shell electrons from other through sp^3^-hybridized orbitals [62]. However, this structure results in the remaining lone-pair electrons being easily oxidized in the air, which seriously affects BP properties and induces degradation of device performance. As shown in Fig. 2A, the distance between neighboring BP layers is only 0.53 nm, so that the single or fewer layers of BP film can be obtained from bulk crystal by mechanical or liquid exfoliation [63,64]. The monolayer folded along the x-direction contains two types of chemical bonds. The longer bond connecting two P atoms between the top and bottom in a monolayer has a bond length of 0.2244 nm. Another shorter bond connecting the two P atoms in the same plane has a bond length of 0.2224 nm [65,66]. From the z-direction view, the BP lattice displays a hexagonal structure with two types of bond angles. The smaller bond angle of 96.3°consists of 1 short bond and 1 long bond, and the larger bond angle of 102.1° consists of two short bonds (Fig. 2B). Thickness dependence of the BP bandgap is definite: it monotonically increases from a narrow bandgap (about 0.3 eV) for bulk to a large gap (about 2 eV) for monolayer, owing to the stronger coupling of interlayer electronic state contrast to other 2D materials [67]. The widely tunable and direct bandgap from the bulk down to monolayer make BP particularly suitable for efficient photodetection and light emission. Several researchers have experimentally measured and theoretically demonstrated the thickness-dependent of BP by IR, photoluminescence (PL), and scanning tunneling spectroscopy [28,42,64,68–74], as shown in Fig. 2C. By alloying BP with arsenic, Liu et al. [75] reported a black arsenic-phosphorus (b-As_x_P_1-x_) with tunable bandgaps in the range of 0.3 to 0.15 eV, confirming that the bandgap of the BP can be further modified.

The key characteristic that distinguishes BP from other 2D materials is unique anisotropy, which is evident in its crystal structure [76,77]. The zigzag and armchair directions are parallel and perpendicular to the atomic ridge, respectively. Strong structural anisotropy caused by these two inequivalent orientations in the BP lattice is the reason that makes its unique in-plane anisotropy for thermal, electrical, and optical characteristics. Angle resolved absorbance, Raman spectroscopy, and PL spectra are commonly method to reveal BP optical anisotropic properties. Ling et al. [78] demonstrated the in-plane optical anisotropy of BP samples through angle resolved absorbance. The electron–phonon interaction is related to the crystalline orientation of BP samples. Mao et al. [79] utilized polarized optical microscopy to research optical anisotropy of few-layer BP under the white light illumination, as shown in Fig. 2D. The evidence shows that the difference between the real and imaginary refractive indices along two directions is what causes optical anisotropy of BP. Wu et al. [80] identified orientation of BP samples using Raman spectroscopy. When the incident light is parallel to the armchair (or zigzag) direction, the local maximum (or minimum) of Ag^2^ mode intensity is achieved. Alodan et al. [81] observed highly anisotropic PL of BP nanosheets in the wavelength range from 590 to 720 nm.

## BP for Photodetection

**Fig. 3.**
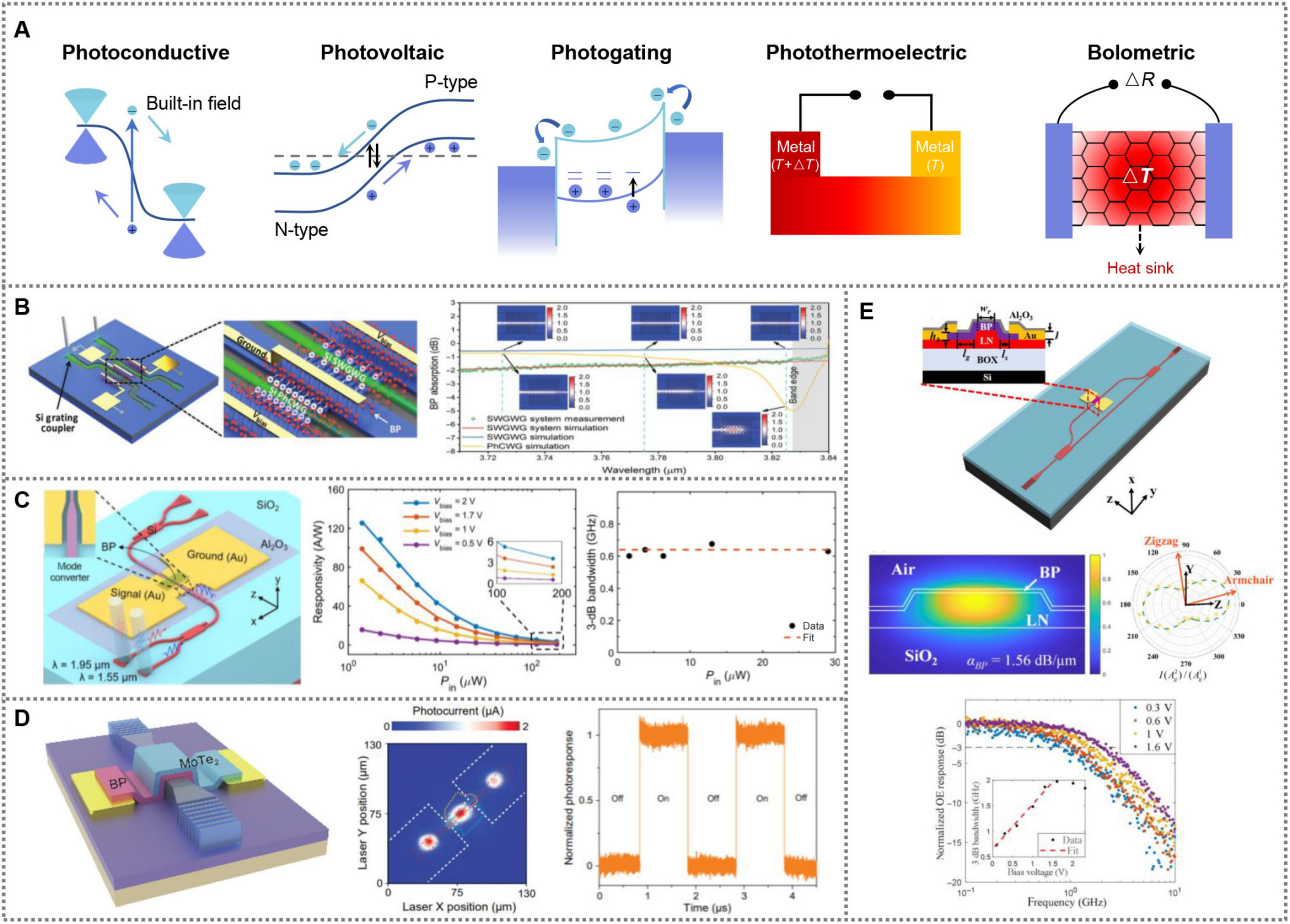
(A) Different photocurrent generation mechanisms: photovoltaic effect, photogating effect, photothermoelectric effect, and bolometric effect. (B) A slow-light-enhanced photodetector based on the PhC WG for mid-IR. Reproduced with permission from [89]. Copyright 2020 Wiley-VCH. (C) A novel avalanche photodetector based on the BP/silicon hybrid plasmonic waveguide operating at wavelengths of 1.55 and 1.95 μm. Reproduced with permission from [90]. Copyright 2022 American Chemical Society. (D) A mid-IR photodetector based on the BP/MoTe_2_ van der Waals heterostructure. Reproduced with permission from [91]. Copyright 2022 American Chemical Society. (E) An integrated BP photodetector based on the TFLN waveguide. Reproduced with permission from [92]. Copyright 2022 Optica Publishing Group.

Photodetectors, as the devices that convert light into electrical signal, have attracted immense research interest in many fields. BP has been demonstrated to be a promising 2D semiconductor for visible to mid-IR photodetectors due to its unique properties. The wide spectral responsivity offers a new opportunity for broadband photo detection [82]. The BP exhibits great differences in carrier mobility and optical absorbance along the zigzag and armchair directions due to strong in-plane anisotropy. The increase of BP thickness also greatly improves the optical absorption. This indicate that the crystalline orientation and thickness of the BP play crucial roles on the responsivity and bandwidth of the photodetectors. [83–85]. There are two categories of mechanisms for 2D materials photodetection, the photon-related detection mechanisms (including photoconductive effect, photovoltaic effect, and photogating effect) and thermal-related mechanisms (including photo-thermoelectric effect and bolometric effect), as shown in Fig. 3A. The photoconductive effect and photovoltaic effect serve as primary mechanisms for integrated BP photodetector. The incident photons induce electron-hole pairs and increases carrier concentration and conductivity in the semiconductor material. With an applied bias voltage, the photogenerated electron-hole pairs are separated and move respect to the electric field, and then comes photocurrent generation by photoconductive effect [86]. For photovoltaic effect, photogenerated electron-hole pairs are separated according to the electric fields in the semiconductor depletion region. The intrinsic electric field is usually generated by the noticeable difference of work function between two materials, such as p-n junctions and Schottky barrier junctions [87]. Despite the photon-related detection mechanisms systematically described above, to realize high responsivities and large bandwidths simultaneously remains a huge challenge. Researchers proposed various structures to increase the responsivity of BP-based photodetectors.

Youngblood et al. [88] utilized few-layer BP to design a waveguide-integrated and gate-tunable photodetector for near-IR telecom band. The performance of the BP photodetector is optimized by integrating a field-effect transistor on the waveguide to induce doping on the BP layer. Compared with graphene counterparts, the dark current in BP photodetectors is drastically decreased when the BP are of low doping, where the photovoltaic effect dominates. Additionally, the device exhibits an excellent responsivity of 657 mA/W at room temperature using a 100-nm thickness of BP.

Further miniaturization of the photodetector increases responsivity, response speed, and internal quantum efficiency. However light–matter interaction lengths are relatively reduced, which limits the BP light absorption. In order to enhance the responsivities in integrated-waveguide BP photodetectors, Ma et al. [89] designed a slow-light-enhanced Mid-IR photodetector on the photonic crystal (PhC) waveguide (WG) with a length of 10 μm, as shown in Fig. 3B. The enhancement of the slow light is verified and characterized through the shared-BP photonic systems including two BP photodetectors fabricated by a PhC WG and a spatially close subwavelength grating waveguide, respectively. The photocurrent is dominated by the photoconductive effect. The BP photodetector based on the PhC WG exhibits a high responsivity (11.31 A/W) and a low noise-equivalent power (0.012 nW Hz^−1/2^) at a bias voltage ~0.5 V.

Liu and co-workers [90] reported a high-responsivity avalanche photodetector based on the plasmonic enhanced BP integrated on silicon waveguide at wavelengths of 1.55 and 1.95 μm, as shown in Fig. 3C. This device consists of two nanoslots between the ultrathin silicon waveguide at the middle and two metal contacts on both sides. This waveguide structure not only facilitates the light–BP interaction but also reduces carrier transit time. Owing to the photovoltage effect in BP, the avalanche photodetector achieves a high responsivity of 125 A/W at the wavelength of 1.95 μm and a 3-dB bandwidth of 1.05 GHz.

Another strategy is to use 2D van der Waals heterostructures to enable photodetection with high dark current suppression. As shown in Fig. 3D, Chen et al. [91] reported a mid-IR photodetector using BP/MoTe_2_ van der Waals heterostructure to realize high-performance photodetection. Scanning photocurrent mapping demonstrates that the photovoltaic response mainly derived from the BP/MoTe_2_ heterojunction rather than from the Schottky junction. They reported response and recovery of ∼30 and ∼58 ns under the laser illumination of ~6.7 μW nm, which are much faster than those of In_2_Se_3_-based photodetectors.

The thin-film lithium niobate (TFLN) platform provides a new way for photonic integrated circuits, attributing to excellent crystalline properties and optoelectronic features of TFLN. As shown in Fig. 3E, Xue et al. [92] reported a high-speed integrated BP photodetector based on the TFLN waveguide. The trap-induced photoconductive gain is considered as dominant photoresponse mechanism. Benefiting from the higher index contrast, the integrated-waveguide BP photodetector exhibits a high absorption coefficient of 1.56 dB/μm and a high responsivity of 2.64 A/W. A 3-dB bandwidth of 1.97 GHz has also been measured.

We summarize various integrated BP-based photodetectors, and their performance metrics are highlighted in Table 1 [82–85,88–92]. For gate-tunable photodetector, low dark current can be realized by integrating a field-effect transistor on the waveguide to induce low doping. Benefiting from the enhanced light–matter interactions, PhC WG, chalcogenide glass waveguide, and TFLN waveguide significantly enhance the responsivity of photodetectors. Furthermore, heterostructures formed by BP and other 2D materials not only increases the response speed but also reduces dark current.

**Table 1.** A comparison of the various BP photodetector metrics.

Structure	Wavelength (nm)	*T*_BP_(nm)	Responsivity(A/W)	3-dBBandwith(GHz)	Detection mechanisms	Reference
Al_2_O_3_/BP/SiO_2_/P-Si	400–3,800	30	6	N/A	Photoconductive	[82]
BP/ChG waveguide	2,185	8.3	0.04	N/A	Photovoltaic	[83]
Al_2_O_3_/BP/SOI	3,680	40	23	N/A	Photoconductive	[84]
Al_2_O_3_/BP/SOI	2,000	40	0.3067	1.33	Photovoltaic	[85]
BP/SOI	1,570–1,580	100	0.657	2.8	Photovoltaic	[88]
BP/PhC WG	3,825	40	11.31	0.00055	Photoconductive	[89]
BP/Al_2_O_3_/SOI	1,550–1,950	30	125	1.05	Photovoltaic	[90]
BP/MoTe_2_/SOI	3,000–3,900	25	0.85	N/A	Photovoltaic	[91]
Al_2_O_3_/BP/TFLN	1,550	39.8	2.64	1.97	Photoconductive	[92]

N/A, not applicable; ChG, chalcogenide glass.

## BP for Optical Modulation

The performance of optical modulators can be characterized by several parameters including modulation speed, modulation depth, and energy consumption. These parameters can be regulated by applied external fields such as electric fields, optical fields, and magnetic fields. Although relative higher modulation depth can be easily realized by electro-optical modulation, the energy consumption and modulation speed are still limiting factors. All-optical modulation without electro-optical conversion increases the modulation speed and realizes lower power consumption with larger bandwidth. However, the modulation depth still needs to be improved [93]. Magneto-optic modulation is realized by using external magnetic field to control optical response, has received as less attention as electro-optical or all-optical method modulation, but could be useful for deep ultraviolet applications [94].

### All-optical modulation

**Fig. 4.**
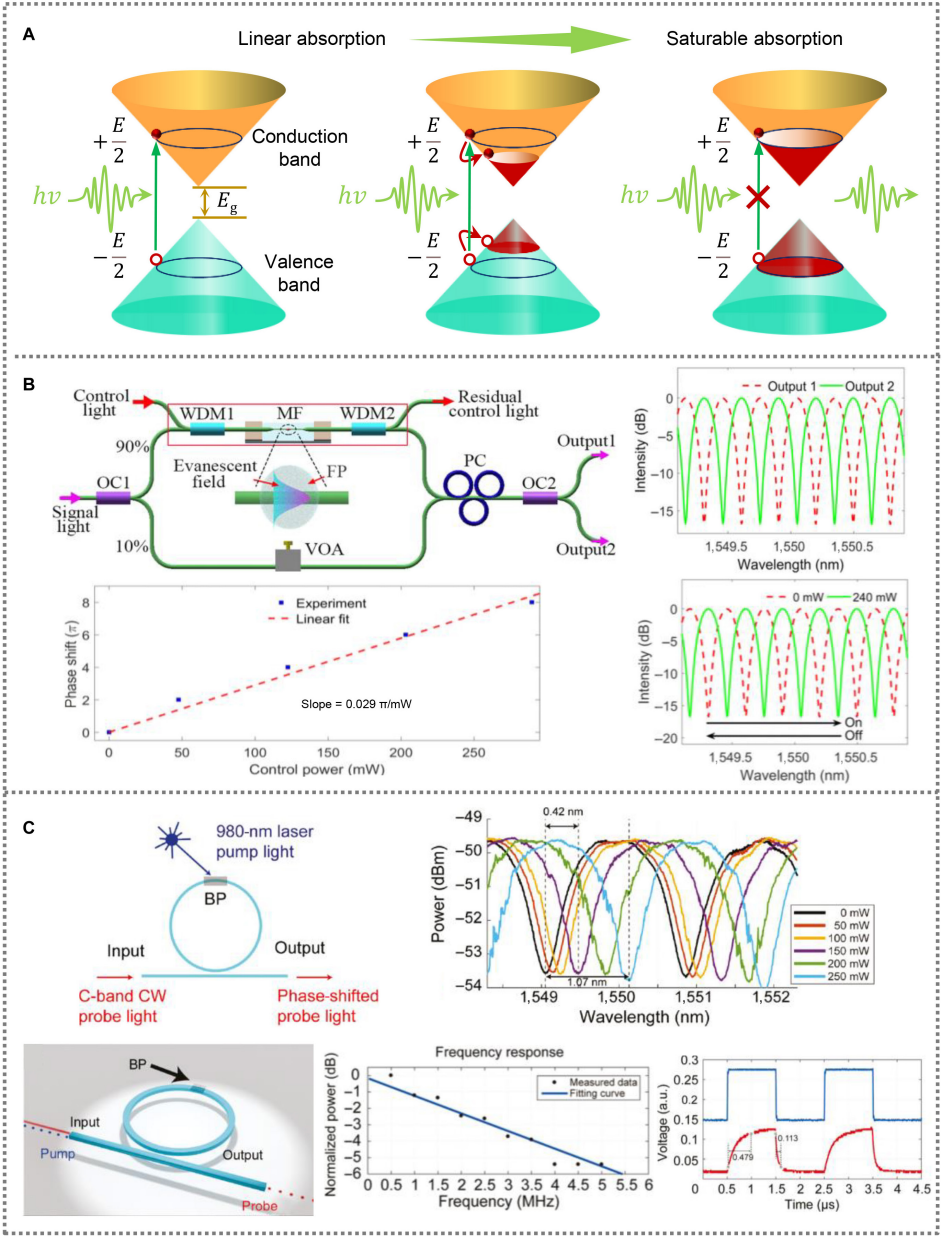
(A) Schematic for saturable absorption. (B) A fiber-based all-optical modulator based on a Mach–Zehnder interferometer structure consisting of few-layer fluorinated phosphorene. OC1 and OC2, two optical fiber couplers; WDM1 and WDM, two wavelength division multiplexers; MF, microfiber; VOA, variable optical attenuator; PC, polarization controller; FP, fluorinated phosphorene. Reproduced with permission from [99]. Copyright 2017 Wiley-VCH. (C) An integrated all-optical modulator based on BP thin film and micro-ring resonators. CW, continuous wave. Reproduced with permission from [100]. Copyright 2020 Walter de Gruyter Foundation.

Optical modulators are devices that tune the amplitude or phase of the light by applying light or electric field. All-optical modulators play a crucial role in high-speed photonic systems. BP is a broadband saturable absorbing material at the wavelengths from visible to mid-IR, which can be used in all-optical modulation and pulsed light generation [95,96]. Saturable absorption is a nonlinear optical phenomenon caused by Pauli-blocking effect, which occurs under high intensity of light [97]. As shown in Fig. 4A, when the energy of the incident light is greater than the bandgap of the material, photons can be absorbed and, in turn, electrons in the valence band are excited into conduction band. As the light intensity increases to a relatively high level, the number of the photocarriers increases above the threshold. At the same time, the conduction band cannot accept any more incoming electrons and the absorption is reduced [98].

Wang et al. [99] demonstrated a novel all-optical BP modulator with a fiber Mach–Zehnder interferometer structure, as shown in Fig. 4B. To improve its long-term environmental stability, BP was prepared by an electrochemical exfoliation method and then simultaneously fluorinated in an electrochemical exfoliation. After reaction, few-layer fluorinated phosphorene was deposited onto a microfiber using optical deposition approach. When the controlling light is coupled into fluorinated-phosphorene-deposited microfiber, the fluorinated phosphorene could absorb the light and increase surface temperature, which results in a phase shift in signal light. The maximum phase shift of the phase modulator is 8π, and the maximum interference contrast is 17 dB with a control light power of 290 mW.

In order to enhance the performance of all-optical modulators, integrating the saturable absorber materials on silicon waveguide is an effective way. As shown in Fig. 4C, Cheng et al. [100] integrated a BP thin film on silicon micro-ring resonators, realizing a BP-integrated all-optical modulator. BP with a thickness of 22 nm was exfoliated and transferred onto the ring resonator by a transfer print method. The temporal and frequency responses of the modulator were observed when both the pump and the probe light were coupled into the waveguide. Based on the photo-thermal effect, the BP-integrated all-optical modulator exhibited better performance including faster response time (rise time of only 479 ns and decay time of 113 ns), broader 3-dB bandwidth (about 2.5 MHz), and higher tuning efficiency (0.164 π/mW), compared to fiber-based all-optical modulators. This work established an alternative strategy for developing integrated all-optical modulators.

### EO modulation

EO modulator is one of the most important components in photonic integrated circuits. It has advantages over other types of modulators in terms of optical loss, power consumption, and speed [101]. Electrorefractive modulators tune the real part of the refractive index by applying an electric field. On the contrary, the electroabsorptive modulators tune the imaginary part of the refractive index by applying an electric field.

**Fig. 5.**
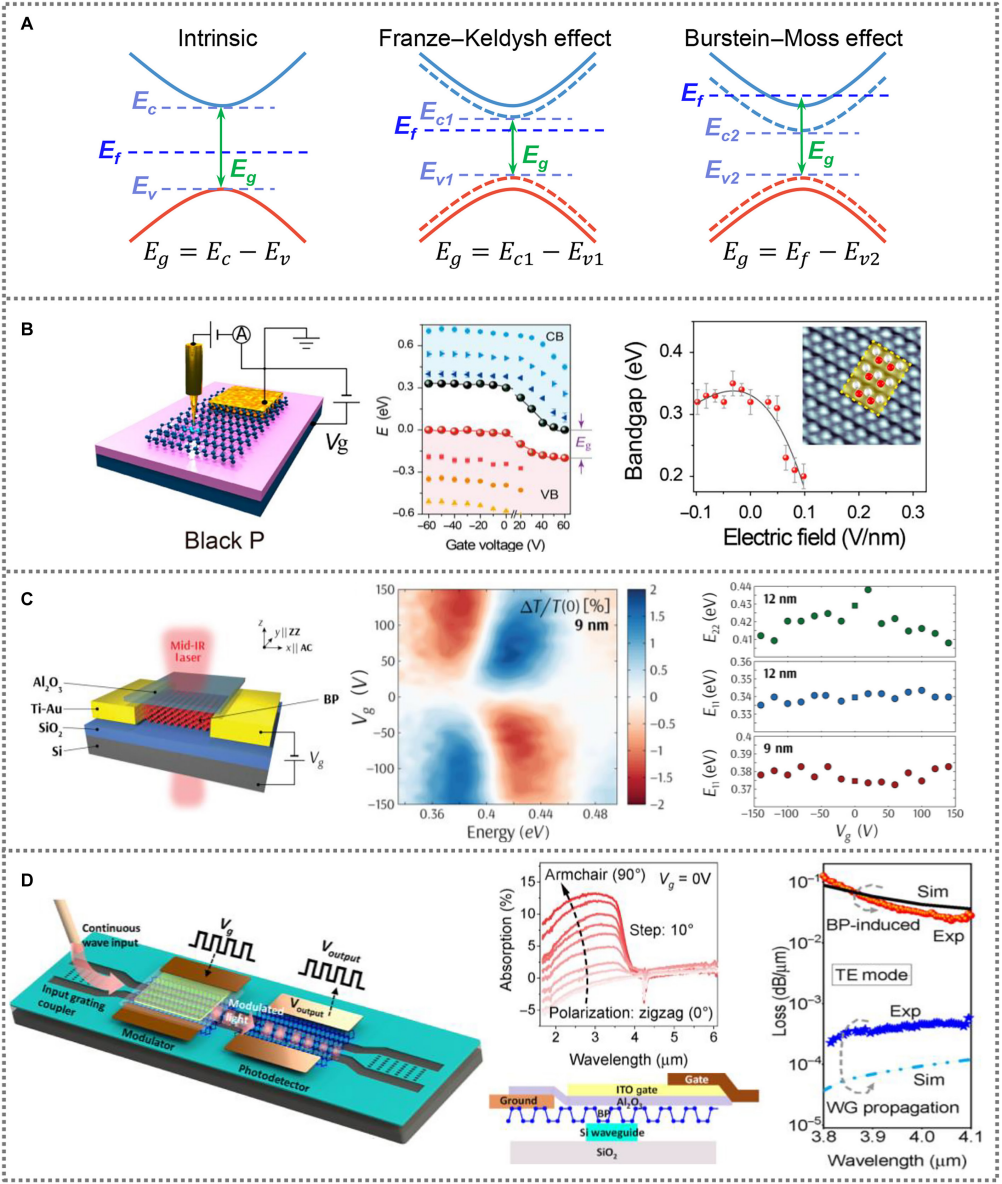
(A) Schematic for Franze–Keldysh and Burstein–Moss effect. (B) Stark effect in electrostatically gated few-layer BP observed by low-temperature scanning tunneling microscopy. Reproduced with permission from [105]. Copyright 2017 American Chemical Society. (C) An EO modulator with multilayer BP for the mid-IR range. Reproduced with permission from [106]. Copyright 2017 American Chemical Society. (D) Hybrid integration of mid-IR EO modulator on silicon waveguide. TE mode, transverse electric mode. Reproduced with permission from [108]. Copyright 2021 Elsevier.

The EO modulation in BP is dominated by quantum constrained Franz–Keldysh effect and Burstein–Moss effect, as shown in Fig. 5A. With an out-of-plane electric field, the electronic bands in BP exhibit a relative bending. It brings valence band maximum and conduction band minimum closer. The reduction of effective bandgap facilitates BP to absorb photon energies with broader range. This modification of BP optical response can be described by the quantum-confined Franz–Keldysh effect, enabling efficient EO modulations [102]. The Burstein–Moss effect is a change in bandgap of the semiconductor resulting from band filling. By applying an external gate bias voltage, the increase of charge carrier density reduces the available unoccupied electronic states. This leads to a decrease in optical conductivity at the transition energy [103,104]. Unlike the quantum-confined Franz–Keldysh effect, the Burstein–Moss effect broadens the effective optical bandgap in BP.

Using scanning tunneling microscopy, Liu et al. [105] observed bandgap reduction in BP flakes, induced by a giant Stark effect by electrostatic gating, as shown in Fig. 5B. Experimental results show that BP bandgap monotonically reduces with increasing electric field intensity, which is due to band filling. In an 11-layers BP flake, the bandgap shrinks to 75% when applying a field intensity of 0.1 V/nm.

Peng et al. [106] reported a mid-IR BP EO modulator using a silicon substrate as a back gate with normal incident light, as shown in Fig. 5C. They further studied its EO modulation ability by taking advantage of the quantum confined Franz–Keldysh effect. The EO modulation exhibits clear dependence on the BP thickness and gate voltage. Strong intensity modulation can be observed at different photon energies of 0.38, 0.43, and 0.5 eV when photon energy are continuously scanned. In addition, a strong layer correlation is also observed by comparing 9- and 12-nm-thick BP samples. The two modulation mechanisms need to be further studied. Lin et al. [107] reported that operating BP in the Burstein–Moss effect and quantum-confined Franz–Keldysh effect can greatly improve electro-absorption as well as power efficiency. The relative contribution of the Burstein–Moss effect and quantum-confined Franz–Keldysh effect is related to the doping range and multilayer BP thickness.

To further improve the extinction ratio, Huang et al. [108] integrated BP on silicon waveguide to construct an EO modulator for the mid-IR ranging from 3.85 to 4 μm, as shown in Fig. 5D. The signal is modulated by gating the BP with a top gate by a combination of the Burstein–Moss and Franz–Keldysh effects. The modulation depth of ~5 dB is achieved at a bias voltage of −4 V with a footprint of 225 μm^2^. The 3-dB bandwidth of the modulator exhibits an upward trend until it saturates at around 400 kHz with the increase of wavelength from 3.85 to 4.1 μm, which would be sufficient for sensing requirement of on-chip mid-IR systems.

We compare various BP-based EO modulators, as shown in Table 2 [106–108]. The Burstein–Moss effect and quantum-confined Franz–Keldysh effect improve the modulation depth efficiently.

**Table 2.** A comparison of the various BP EO modulator metrics.

Structure	Wavelength (nm)	*T*_BP_(nm)	Modulation depth(dB)	Modulation mechanisms	Reference
Al_2_O_3_/BP/SiO_2_/Si	2,500–3,700	9	5	Franz–Keldysh	[106]
Al_2_O_3_/BP/SOI	3,300	20	N/A	Burstein–Moss and Franz–Keldysh	[107]
ITO/Al_2_O_3_/BP/SOI	3,850–4,100	20	5	Burstein–Moss and Franz–Keldysh	[108]

## BP for Light Source

Light emitter is widely used in the field of communication [109], sensing [110], and medical treatment [111]. The operating wavelength of a light emitter depends on the bandgap of the material. Similar to the BP-based photodetectors, the light emitters also display strongly anisotropic properties. The polarized emission along the armchair and zigzag crystal direction reaches maximum and minimum, respectively. The tunable bandgap makes BP a potential light-emitting material in constructing multifunctional IR light emitters [112,113].

**Fig. 6.**
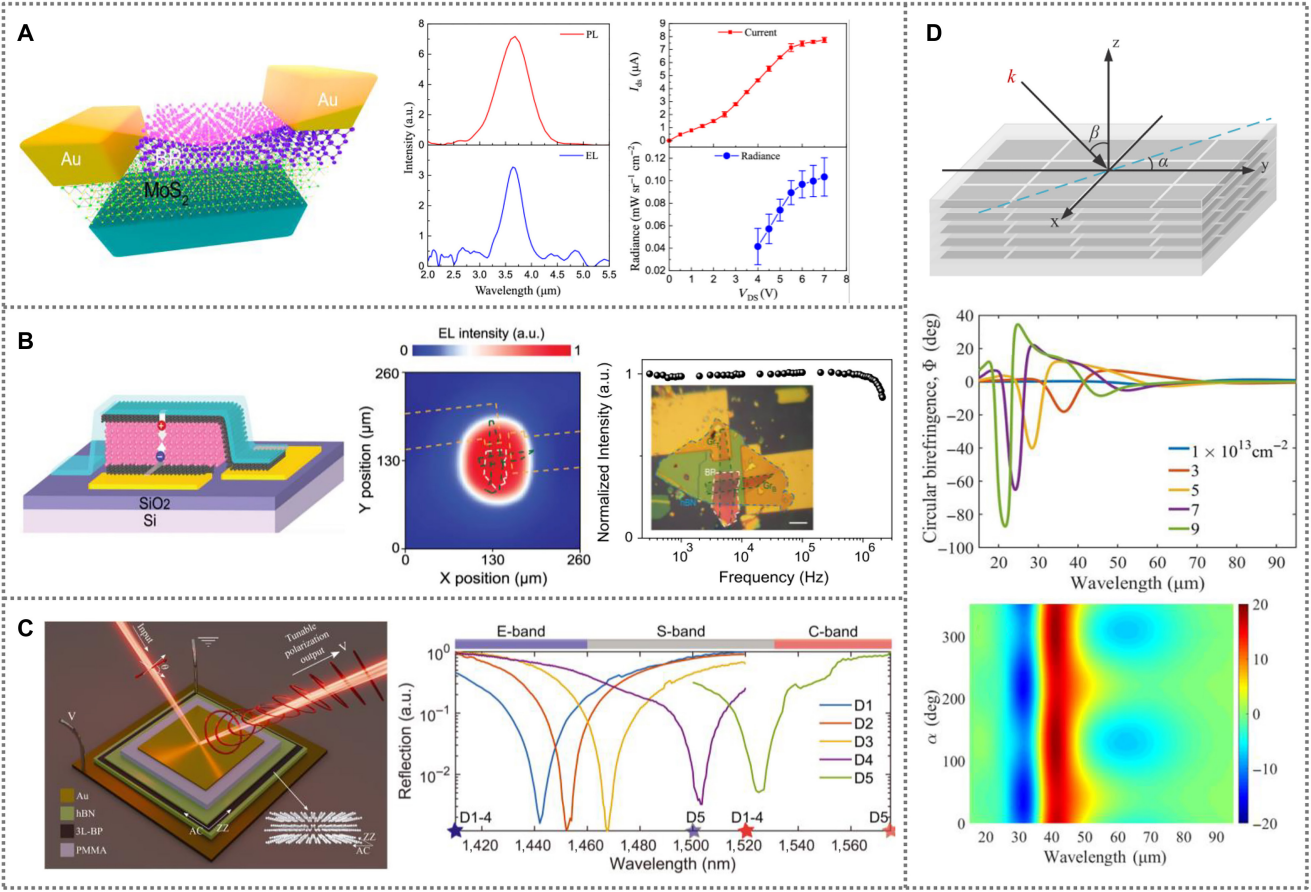
(A) A mid-IR light-emitting diode based on the BP/MoS_2_ heterojunction. Reproduced with permission from [114]. Copyright 2020 American Chemical Society. (B) A mid-IR light-emitting diode based on the hBN/Gr_T_/BP/Gr_B_ heterostructures. Reproduced with permission from [115]. Copyright 2020 American Chemical Society. (C) A trilayer BP heterostructure integrating in a Fabry–Pérot cavity for polarization conversion. Reproduced with permission from [126]. PMMA, polymethyl methacrylate. Copyright 2022 American Association for the Advancement of Science. (D) A twisted multilayer BP structure and its optical responses to circularly polarized light. Reproduced with permission from [128]. Copyright 2022 Optica Publishing Group.

Wang et al. [114] proposed a room-temperature light-emitting diode based on the BP/MoS_2_ heterojunction prepared by mechanically exfoliated method (Fig. 6A), showing polarized electroluminescence (EL). Under a forward bias voltage of *V_DS_* = 7 V, the EL has a wavelength of 3.68 μm, and the internal quantum efficiency and external quantum efficiency are calculated to be 0.03% and 1%, respectively.

Chang et al. [115] integrated a BP mid-IR light emitter on silicon waveguide using graphene as transparent contacts, as shown in Fig. 6B. Under forward bias, the hBN/Gr_T_/BP/Gr_B_ heterostructures light-emitting diode emits polarized EL at *λ* = 2.5 μm and λ = 3.5 μm, with external quantum efficiencies of 80% and 0.084%, respectively.

Owing to the actively tunable bandgap property, Kim et al. [116] demonstrated room-temperature tunable IR BP light-emitting diodes by straining the substrate. PL spectroscopy shows that the PL intensity increases with the enhancement of material strain, and the EL increases with the PL quantum yield. The internal quantum efficiency of BP light emitter varies from 0.73% (0.2%; compressive) to 2.47% (1.0%; tensile) due to its highly strain-sensitive nature.

Interestingly, the peak position of the EL and PL spectra both shows an anomalous blueshift with increasing temperature [117,118]. This phenomenon can be attributed to the mechanical strain effect induced by lattice thermal expansion at higher temperatures, which leads to the monotonic increase of the BP bandgap with temperature. The temperature dependence of BP provides a promising strategy for tuning waveguide-integrated BP-based light emitters.

Overall, scientists proposed various light emitters based on BP with different structures, and their key parameters are summarized in Table 3 [114–120]. In addition to the in-plane anisotropy and thickness dependency, the temperature dependency and strain dependency of BP are the key factors affecting performance of the light emitters. Light emission can be greatly improved by integrating BP or its heterostructure on the integrated photonic structures, such as Al_2_O_3_/Au optical cavities or silicon photonic waveguides.

**Table 3.** A comparison of the various BP light emitter metrics.

Structure	Wavelength (nm)	*T*_BP_(nm)	Internal quantum efficiencies (%)	Eternal quantum efficiencies (%)	Reference
BP/MoS_2_	3,680	70	0.03	1	[114]
hBN/Gr_T_/BP/Gr_B_	3,500	65	N/A	0.084	[115]
BP/MoS_2_/PETG	5,500	20	0.73–2.47	N/A	[116]
hBN/BP	2,800–4,000	46	N/A	N/A	[117]
BP/WSe_2_	2,790	6	N/A	N/A	[118]
BP/Al_2_O_3_/Au	3,650	80	N/A	4.43	[119]
BP/MoS_2_/b-As_0.46_P_0.54_	2,500–6,000	20	N/A	0.3	[120]

## BP for Polarization Conversion

The anisotropic crystal structure makes BP a competitive candidate for polarization manipulation [121]. Polarization is one of the fundamental physical features of light and is widely used in many fields [122–125]. BP has excellent electrically tunable optical dichroism, which can directly respond to polarized light, ensuring the development of polarization-sensitive optoelectronic devices.

Biswas et al. [126] proposed a reconfigurable polarization converter by integrating the trilayer BP onto an optically resonant cavity for telecommunication wavelengths ranging from 1,410 to 1,575 nm. The cavity is formed between a high-reflectivity gold mirror and a partial-transmitting thin gold mirror, as shown in Fig. 6C. The trilayer BP is encapsulated by hexagonal boron nitride, and different thickness of polymethyl methacrylate is used to adjust the cavity resonance frequency. The polarization conversion spans nearly half the Poincaré sphere by electrical tuning. Matthaiakakis et al. [127] reported an approach to realize tunable polarization conversion without asymmetric and chiral nanostructure. The dynamic polarization conversion from linearly polarized light to circularly polarized light can be achieved by altering ellipticity of the scattered wave. Hu et al. [128] proposed a multilayer twisted BP stack, as illustrated in Fig. 6D. Circular dichroism and circular birefringence responses are studied. The twisted BP stacks make it possible to enable giant tunability in polarization.

The polarization conversion based on the BP has been made progress in structure design and integration method. However, how to further improve the dynamic range and active control are problems to be solved.

## Conclusion

Photonic integrated circuits are driven by the increasing requirements in bandwidth of communication networks. The potential for low energy consumption boosts its importance as an economical and effective solution compatible with electronics. The development of the optoelectronic devices is oriented toward large-scale integrated chips, and BP-integrated photonic circuits are considered to be a strategy to increase the integration level. The unique optical properties of BP offer new opportunities and promote new applications. We review the main applications of BP-integrated photonics including photodetection, modulation, light emission, and polarization conversion.

The BP-integrated photodetector exploits different photoelectric effects and provides wide operation bandwidth from visible to mid-IR on various substrates to match different application requirements. As for BP modulators, more efforts are needed to improve the electron–photon conversion, modulation efficiency, and response speed. BP light emitters have been demonstrated recently. The optimization of the device geometry and structure is an effective approach to enhance light–matter interaction and thus increasing efficiency. BP for polarization conversion is new and provides a new strategy to manipulate polarization in the mid-IR. The degradation of BP due to oxygen, water, and light is no longer a limiting factor since it could be effectively prevented by encapsulation and packaging.

Overall, BP-integrated photonic circuits have attracted considerable attention, and many advances have been achieved in diverse aspects, such as theoretical study, material synthesis, integration methods, encapsulation strategy, and structural design. BP-based photonic devices have considerable potentials, while there are still some challenges for high-performance photonic integrated circuits. Considering the rapid development of synthesis and fabrication techniques, the development of BP-based optoelectronic devices will keep a fast pace.

## Data Availability

The data could be given upon reasonable request from the corresponding authors.
